# Vitamin D mitigates age-related cognitive decline through the modulation of pro-inflammatory state and decrease in amyloid burden

**DOI:** 10.1186/1742-2094-9-244

**Published:** 2012-10-25

**Authors:** Teresita L Briones, Hala Darwish

**Affiliations:** 1 Department of Adult Health, Wayne State University, 5557 Cass Ave., Cohn Bldg, Rm 344, Detroit, MI 48202, USA; 2 Hariri School of Nursing, American University of Beirut, Beirut, Lebanon

**Keywords:** Learning and memory, Object recognition test, IL-1β, IL-10, Aging, Cognitive aging

## Abstract

**Background:**

Increasing evidence shows an association between the use of vitamin D and improvement in age-related cognitive decline. In this study, we investigated the possible mechanisms involved in the neuroprotective effects of vitamin D on age-related brain changes and cognitive function.

**Methods:**

Male F344 rats aged 20 months (old) and 6 months (young) were used and randomly assigned to either vitamin D supplementation or no supplementation (control). A total of n = 39 rats were used in the study. Rats were individually housed and the supplementation group received a subcutaneous injection of vitamin D (1, α25-dihydroxyvitamin D3) 42 I.U./Kg for 21 days. Control animals received equal volume of normal saline. Behavioral testing in water maze and spontaneous object recognition tasks started on day 14. Levels of interleukin (IL)-1β and IL-10 were quantified to assess inflammatory state. Also, beta amyloid (Aβ) clearance and Aβ load were measured.

**Results:**

Our results show that: (1) aged rats demonstrated significant learning and memory impairment overall compared to younger animals. However, the age-related decline in learning and memory was ameliorated by the supplementation of vitamin D. No vitamin D effect on learning and memory was seen in the young animals; 2) the pro-inflammatory cytokine IL-1β is significantly increased while the anti-inflammatory cytokine IL-10 is significantly decreased in the aged rats compared to the young animals; but this age-related change in inflammatory state was mitigated by vitamin D supplementation. No effects of vitamin D were seen on the IL-1β and IL-10 expression in the young rats; (3) vitamin D increased Aβ clearance and decreased amyloid burden in the aged rats while no significant difference was seen between the young animal groups.

**Conclusions:**

Our data suggest that vitamin D supplementation modulated age-related increase in pro-inflammatory state and amyloid burden. It is possible that these effects of vitamin D mediated the decrease memory impairment seen in the aged rats making it a useful therapeutic option to alleviate the effects of aging on cognitive function.

## Background

Vitamin D deficiency is a concern in adults over the age of 50 years. It is estimated that between 40% and 100% of older, community-living adults in the United States and Europe are vitamin D deficient [1,2]. Vitamin D is a neurosteroid hormone with diverse physiological roles. Evidence shows that the receptors for vitamin D in the central nervous system (CNS) are widely distributed and that the enzyme responsible for the synthesis of the active form of vitamin D is ubiquitous in the brain [3-5]. Based on this information, it is highly likely that vitamin D plays a role in neurological functioning. Indeed, animal studies indicate that vitamin D is important for brain development [6-8]. Additionally, epidemiological studies link the plasma level of vitamin D to a range of brain-related outcomes (reviewed in [4]). Although data in human studies are not consistent, an association between low concentrations of vitamin D and impairments in cognitive functions such as memory and orientation, and executive function is reported, as well as diagnosis of dementia and Alzheimer’s disease [9-14]. Studies in 139 ambulatory nursing home residents with a history of falls and are vitamin D deficient at baseline also report improved attention and reaction times after 6 months of supplementation; and a small improvement in clock drawing performance but not verbal fluency is seen when vitamin D was given over 4 weeks in 25 elderly nursing home participants [15,16]. Other studies suggest the possibility that vitamin D may be neuroprotective because it plays an important role in the expression of neurotrophic factors, neurogenesis, calcium homeostasis, and detoxification [17-20].

Neuronflammation may be an important underlying mechanism in cognitive decline in the elderly as demonstrated by emerging evidence on the association between systemic levels of inflammatory markers to age-related cognitive impairment [21-23]. Neuroinflammation in aging is also implicated in pathological events such as the development of amyloid plaques commonly seen in Alzheimer’s disease [23]. Data from neurocognitive studies in populations of older adults living in the community also consistently show an association between higher inflammatory levels and lower cognitive functioning (reviewed in [24]). Since evidence shows inflammation and vitamin D deficiency is common in aging, and that providing vitamin D supplementation improves age-related cognitive decline, it is possible that the neuroprotective effects of vitamin D involves the modulation of inflammatory state resulting in decreased formation of amyloid beta (Aβ) oligomers, a pathological consequence of chronic inflammation. Thus, in this study we investigated whether vitamin D has the ability to: 1) modulate the exaggerated pro-inflammatory state associated with aging, and 2) reduce levels of Aβ oligomers. In addition, we examined whether the ability of vitamin D to modify these neuropathological processes is associated with improved cognitive functioning.

## Methods

### Subjects

**Figure 1:**
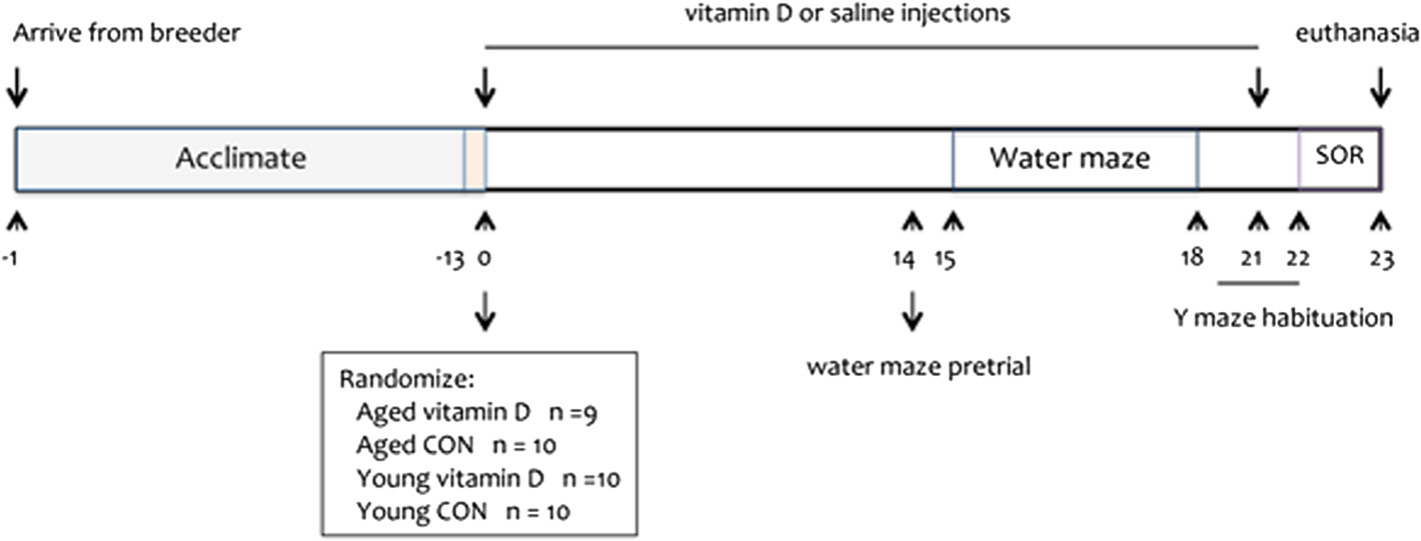
**Study design.** Legend: CON, control; SOR, spontaneous object recognition. Numbers on the horizontal plane represent days.

Male F344 rats age 20 months (aged) and 6 months (young) obtained from Harlan Laboratories (Madison, WI, USA) were used. Rats were housed individually in a pathogen-free vivarium under controlled condition (temperature 22 ± 1°C and humidity 70 ± 5%) and a 14:10 hour light:dark cycle was maintained. All animals were housed in the same room so that temperature, humidity, and lighting conditions are similar for all groups. Animals had free access to food (regular rat chow containing 3 I.U./g vtamin D3, 1.4% calcium, and 1.1% phosphorus) and water. Animals were also handled daily throughout the study so that they could get acclimated to the research personnel thereby decreasing stress. Experiments (Figure 1) started 2 weeks after arrival of the animals and all experimental protocols in this study were approved by the Institutional Animal Care and Use Committee and in accordance with the National Institutes of Health guidelines.

### Vitamin D supplementation

Two weeks after arrival from the breeder, young and aged rats were randomly assigned to either the control (CON) or vitamin D supplementation groups. A total of n = 39 rats were used in the study (n =10 animals in each of the young groups and the aged CON group; n = 9 animals in the aged vitamin D group). The supplementation group received the active metabolite of vitamin D (1, α25-dihydroxyvitamin D3; Calbiochem, LaJolla, CA, USA) 42 I.U./Kg based on our previous work where this amount of vitamin D and duration of supplementation produced an elevation of serum levels without any other side effects (for example, lethargy, weight loss, diarrhea)*.* Vitamin D was prepared daily and dissolved in 1% ETOH (diluted with sterile saline). Injections instead of dietary or water supplementation were chosen to be able to control the amount of vitamin D given because each animal’s dietary and water intake is variable. Rats in the control group received 99% normal saline and 1% ETOH of equal volume to control for the effects of stress induced by the injection. Both vitamin D and normal saline injections were given subcutaneously for a total of 21 days and rats were weighed once a week during the supplementation regimen and no group differences were seen. Rats were also monitored daily for possible side effects (n=0) such as apathy, lethargy, and diarrhea.

### Cognitive testing

Two weeks after vitamin D or saline injections started, rats were tested in the water maze and Y maze to evaluate cognitive impairment (Figure 1, Study design). Both tests assessed learning and memory processes. All testing was done approximately 2 h prior to the onset of the dark cycle to ensure that it is close to the rats’ active period.

### Water maze

Spatial learning and memory (acquisition and recall), tasks sensitive to hippocampal dysfunction were examined using the water maze task as previously described [25,26]. During testing, the water maze tub was filled with tepid water (22 ± 2 C) and made opaque by the addition of powdered milk. The pool was divided into four quadrants of equal surface area and the starting locations for testing were assigned north, south, east, and west. The goal/platform was located in the middle of the southeast quadrant approximately 22 cm from the pool rim. The day before actual testing started, rats were allowed a habituation swim for 10 s without the platform. Animals received four trials a day for 4 consecutive days. During the trials, swim latency (time to reach the platform) and the path taken by the animals to reach the platform were recorded by a video camera connected to an image analyzer (Water Maze System Version 4.20, Columbus, OH, USA). In addition, swimming speed (path length/swim latency) was used to assess the motoric activity in performing the task.

On the last day, a probe trial was performed wherein the animals were tested in the water maze but the goal/platform was removed. Measures evaluated in the probe trial were: amount of time spent in the correct quadrant where the goal/platform was previously located, and search errors, which represent the number of entries made to incorrect quadrants in the pool. An error was designated only if the rat’s entire body (excluding the tail) is in an incorrect quadrant.

### Y maze

**Figure 2:**
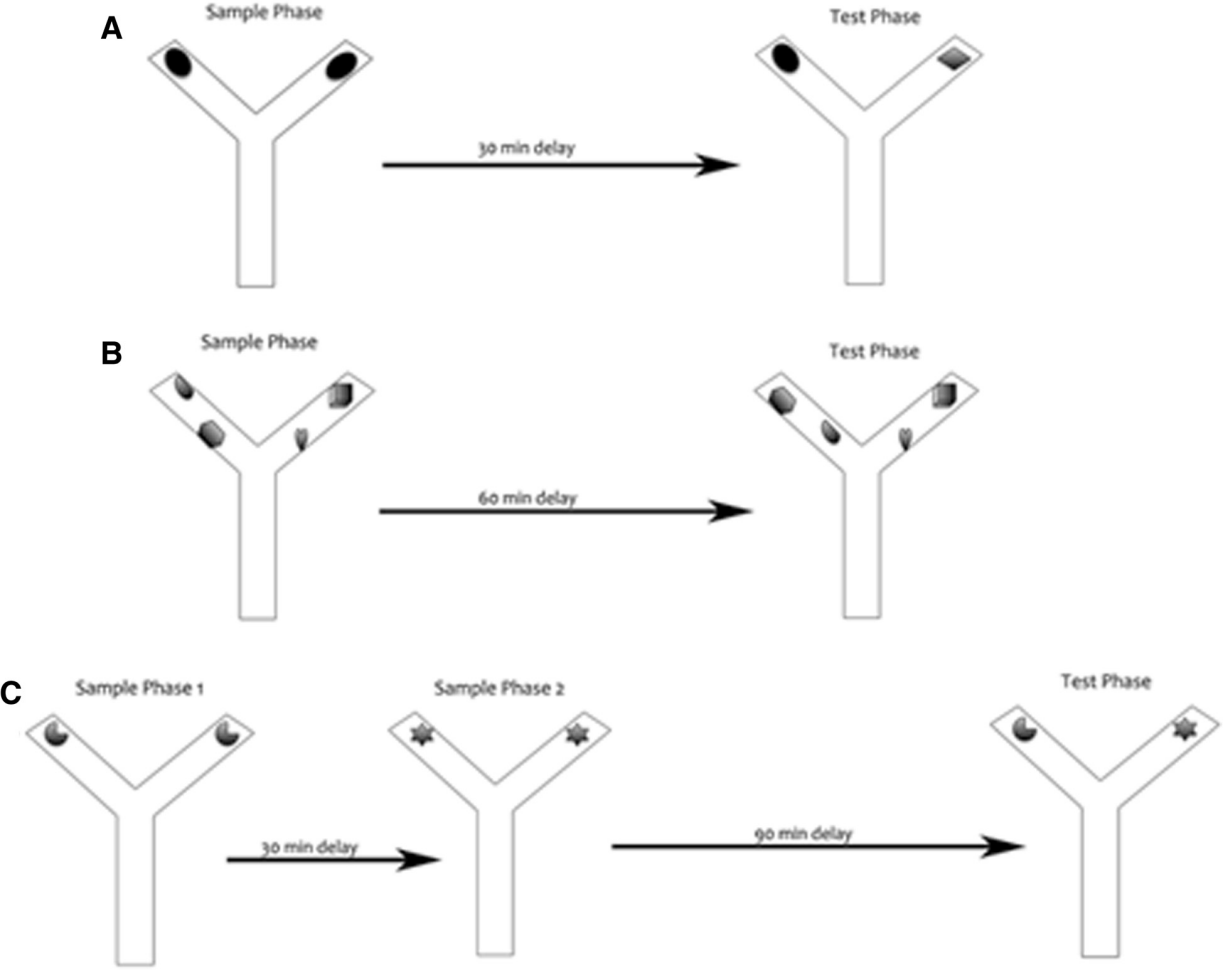
**Spontaneous object recognition tasks.** Diagrams of the three spontaneous object recognition tasks used in the study: novel object recognition (**A**), object-in-place test (**B**), and temporal order memory (**C**).

Spontaneous object recognition and temporal order memory were tested using the Y maze. Rats were habituated to the Y maze without any objects for 1 min per day for 3 days to reduce anxiety. Tests in the Y maze consisted of: novel object preference, object-in-place, and temporal order memory tasks (Figure 2). All tests comprised of an acquisition phase (sample phase) and a recognition phase (test phase), separated by a time delay. The maze and objects were wiped with a wet cloth containing sodium hypochlorite solution after each session to eliminate odor cues. Exploratory behavior was defined as the animal directing its nose or sniffing toward the object at a distance of approximately 2 cm. Other behaviors such as looking around while sitting on or resting against the object were not considered exploratory behavior.

The novel object recognition task allows discrimination between novel and familiar objects. This kind of memory consists of two components: recollection, which depends on the hippocampus, and familiarity, which depends on the perirhinal cortex [27]. In the acquisition phase, two identical objects (A1 and A2) were placed near the two diagonal corners of the Y maze and the animals were allowed to explore the objects for 2 min. The delay between the sample and test phases was 30 min. During the test phase, two objects were placed at the same positions as in the sample phase, and one object was the same used in the sample phase (A1), while the other was a novel object (B). The time spent exploring the novel and familiar objects was recorded for 2 min but attention was focused on the first minute, during which rats’ preference for the novel object is typically greatest [28]. The position of the objects in the test phase and the objects used as novel or familiar were counterbalanced between rats.

The object-in-place task was started 2 h after completion of the novel object recognition test. In this task, the rat’s ability to recognize objects it had experienced before that remained in the same location versus those that had changed location was assessed. In the acquisition phase, the rat was exposed to objects C1 and C2, and D1 and D2, which were placed in the Y maze as in the novel object recognition test and the animal was allowed to explore all objects for 2 min. After a delay of 60 min, the test phase began in which objects C1 and C2 remained in the same position but objects D1 and D2 were moved or placed in different locations. Thus, all objects in the test phase were equally familiar, but two were in new locations and the other two remained stationary. The objects moved and the positions of the objects were counterbalanced between rats. If object-in-place memory is intact, the animal should spend more time exploring the two objects that are in different locations compared to the two objects that are in the same locations.

Temporal order memory task was started 24 h after completion of the object-in-place test. This task comprised of two sample phases and one test trial. In each sample phase, the animals were allowed to explore two copies of an identical object for a total of 2 min. Different objects were used for sample phases 1 and 2, with a delay between the sample phases of 30 min. The test trial (2 min duration) was given 90 min after sample phase 2. During the test trial, a copy of the object from sample phase 1 and a copy of the object from sample phase 2 were used. The positions of the objects in the test and the objects used in sample phases 1 and 2 were counterbalanced between the animals. If temporal order memory is intact, the rats should spend more time exploring the object from sample 1, that is, the object presented less recently, compared with the object from sample 2, that is, the new object.

In all the tasks, the rats were placed in the Y maze start box and the guillotine door was opened to allow the rat entry into the main area of the apparatus; the door was closed immediately once the rats had vacated the start box to prevent re-entry into this area. Timing of the exploration session did not begin until the rat had exited the start box. The Y shape maze was used to reduce the spatial and contextual information and test only object recognition abilities of the animals. All sessions (acquisition and testing) were taped and exploration time was calculated after the sessions were completed**.** The total time exploring each object and the discrimination ratio, which is the difference in time exploring novel and familiar objects divided by the total time were calculated.

### Tissue preparation

All rats were euthanized using CO_2_ asphyxiation after behavioral testing, the brains removed, cut in half sagitally and both hippocampi manually dissected then immediately placed in liquid nitrogen and kept frozen until processed. One half was used for real-time polymerase chain reaction (RT-PCR) for determination of cytokines IL-1β and IL-10 messenger RNA (mRNA) levels and the other half was used for western blot (Aβ oligomers, BACE1, and neprilysin levels) and ELISA (IL-1β and IL-10 protein expression).

### Real-time polymerase chain reaction

To examine mRNA expression of IL-1β and IL-10, RT-PCR was used. After 0.1 g of frozen hippocampal tissue was homogenized in Trizol (Invitrogen, Carlsbad, CA, USA), total RNA was extracted using an RNeasy extraction kit (Qiagen, UK) according to the manufacturer’s protocol. From the RNA, cDNA was synthesized with oligo primers (listed in Table 1) at 50°C using the SuperScript III First-Strand Synthesis (MBI Fermentas, Glen Burnie, MD, USA) following the manufacturer’s instructions. After reverse transcription, the cDNA was diluted 1:4 (for IL-10) or 1:8 (for IL-1β) and the sample was amplified by RT-PCR (LightCycler, Roche Diagnostics, Idaho Falls, ID, USA) using SYBR Green master mix or TaqMan Universal PCR Master Mix (Applied Biosystems, Foster City, CA, USA) and the following cycling parameters were used: initial denaturation and enzyme activation at 95°C for 10 min, followed by 45 cycles of denaturation at 95°C for 15 s, 61°C for 34 s (IL-1β), 60°C for 34 s (IL-10), or 65°C for 30 s (glyceraldehyde-3-phosphate dehydrogenase), and extension at 72°C for 1 min. Copy numbers of IL-1β and IL-10 transcripts were normalized against those of glyceraldehyde-3-phosphate dehydrogenase (GAPDH, housekeeping gene) transcripts for each sample. All reactions were performed in triplicate. For quantitative analysis of target gene mRNA, the comparative threshold cycle (C_*T*_) method was used [29]. The difference between the normalized threshold cycle for the vitamin D and saline samples, as well as the young and aged samples were expressed as fold change.

**Table 1:** Oligo primers

	**Forward**	**Reverse**
IL-1β	5’-AAGCCTCGTGCTGTCGGACC-3’	5’-TGAGGCCCAAGGCCACAGGT-3’
IL-10	5’-GGCATGAGGATCAGCAGGGGC-3’	5’-TGGCTGAAGGCAGTCCGCAG-3’
GAPDH	5’-AGACAGCCGCATCTTCTTGT-3’	5’-CTTGCCGTGGGTAGAGTCAT-3’

Primer sequences for RT-PCR were designed using Applied Biosystem’s Primer Express software.

### Cytokine protein quantification

The concentration of IL-1β and IL-10 protein levels was determined in the hippocampus using commercially available ELISA assays, following the instructions supplied by the manufacturer (R&D Systems; Minneapolis, MN, USA). Briefly, 0.2 g of frozen hippocampal tissue was homogenized with a glass homogenizer in 1 mL buffer containing 1 mmol/L phenylmethylsulfonyl fluoride, 1 mg/liter pepstatin A, 1 mg/liter aprotinin, and 1 mg/liter leupeptin in PBS (pH 7.2), and centrifuged at 12,000 x g for 20 min at 4°C. The supernatant was collected and total protein was determined by bicinchoninic acid (BCA) protein assay reagent kit (PIERCE, Milwaukee, WI, USA). Standards, controls, and samples (50 μL) were pipetted into a 96-well plate precoated with polyclonal antibodies specific for IL-1β and IL-10, then incubated at room temperature for 2 h on an orbital plate shaker (approximately 250 rpm) then washed 5x before adding the conjugate. After several washes, the chromogen (tetramethylbenzidine) was added to each well and incubated for an additional 30 min. Color reaction was stopped by an equal volume of stop solution and reaction was read in a microplate reader (Bio-Tek, Winooski, VT, USA) at a wavelength of 450 nm (650-nm reference wavelength). The color change was proportional to the concentration of the cytokines measured and all samples measured within the range of the standard curve. This ELISA system detects both natural and recombinant rat IL-1β and IL-10. Assays were sensitive to 1.5 pg/mL of IL-1β and 10 pg/mL of IL-10, and inter- and intra-assay coefficients of variation were <10%.

### Western blot

To detect levels of Aβ oligomers, BACE1, and neprilysisn, 0.5 g of frozen hippocampal tissue was used in the western blot procedure. Tissues were homogenized and centrifuged at 25,000 x *g* for 20 min as previously described [25,26]. Aliquots from the supernatant were removed for protein determination. Protein concentration in samples was determined using the BCA-Protein assay (Pierce, Rockford, IL, USA). The primary antibodies used are: (1) anti-BACE1 (1:50, Calbiochem, San Diego, CA, USA) to evaluate βamyloid precursor protein processing; and (2) anti-neprilysin (1:50, Novocastra Laboratories, Newcastle upon Tyne, UK) to evaluate levels of Aβ degrading enzyme.

Western blot procedure was also used to examine formation of Aβ oligomers. After tissue homogenization as described above, some of the samples were removed for extraction of soluble amyloid peptide. Samples were centrifuged at 78,500 x *g* for 1 h at 4°C and the supernatant were removed then processed as described above. Antibody used was anti-A11 (1:200, Biosource, Carlsbad, CA, USA) to determine levels of Aβ oligomers.

For western blot analyses, equal amounts of protein (40 μg) from each rat were loaded and separated by SDS-PAGE gel electrophoresis in 8% to 16% acrylamide gradient gels. The protein bands were electrophoretically transferred to nitrocellulose membranes (Amersham, Piscataway, NJ, USA) stained with 0.5% Ponceau Red to visualize total proteins, then destained. Non-specific binding sites were blocked then nitrocellulose membranes were incubated overnight at 4°C with gentle agitation in the primary antibody**.** The secondary antibodies used are horseradish peroxidase-conjugated immunoglobulin (Sigma, St. Louis, MO, USA) and the Super Signal chemiluminescense substrate kit (Pierce, Rockford, IL, USA) was used to visualize immunoreactive bands. After visualization, the membranes were then stained with Amido-Black to qualitatively verify protein loading. A series of dilutions were performed and immunoblotted for each antibody to establish that the relationship between protein band and intensity was linear over the range of band intensities observed in the samples. Band visualization was obtained by exposure of membranes to autoradiographic film (Kodak Biomax™). Samples were analyzed in quadruplicates and measurements were averaged and used as one individual data point for statistical analysis. Quantification of differences in protein bands between samples was done using densitometric analysis (Scion Image Beta 4.0.2; Frederick, MD, USA). The internal control β-actin was used to standardize experimental values in densitometric analysis. Densitometric values were calculated as: density of sample band/density of background. Values obtained were then converted to percent of young group (approximately 100% ± 3%) since vitamin D did not have any significant effects on BACE1 levels, Aβ clearance, and Aβ burden in these animals.

### Detection of plasma vitamin D levels

Plasma levels of activated 1, α25-dihydroxyvitamin D3 were determined using the commercially available ELISA assay (Immunodiagnostic Systems, Fountain Hills, AZ, USA) according to manufacturer’s instructions. After CO_2_ asphyxiation, the thoracic cavity was opened and blood collected from cardiac puncture was placed in EDTA coated tubes. Samples were centrifuged (6,000 x g for 15 min at 4°C) and stored at 80°C until assaying. Briefly, controls and samples were delipidated and transferred to supplied immunocapsules containing monoclonal antibody to 25(OH)D or 1, α25-dihydroxyvitamin D3 in suspension with vitamin D-binding protein inhibitor for immunoextraction. After eluting calcidiol from the immunocapsule gel, samples were evaporated in borosilicate glass tubes in a heating block for 30 min under nitrogen gas flow. Evaporated samples were reconstituted in assay buffer and incubated overnight with 100 μL of primary antibody solution. Standards, controls, and samples were then pipetted into a 96-well plate precoated with antibodies specific for 1, α25-dihydroxyvitamin D3 then incubated at room temperature for 90 min on an orbital plate shaker. Subsequent steps are similar to those described above for cytokine quantification.

### Statistical analysis

The SAS general linear model (SAS Institute, North Carolina, USA) procedures for two-way analysis of variance (ANOVA) were used to examine effects of experimental conditions (vitamin D vs saline groups) and age (young vs aged) on the dependent variables. Two-way repeated measures ANOVA was used to examine vitamin D supplementation effects on behavioral performance on the water maze to determine differences in swim latency, path length, and swimming speed. When appropriate, the SAS CONTRAST statement was used for planned comparisons of the effects of supplementation (vitamin D vs saline groups) and age (young and aged), and the combination of age and supplementation. All error bars represent ± standard error of the mean (SEM) of the sample size used in the study.

## Results

### Plasma levels of vitamin D

**Figure 3:**
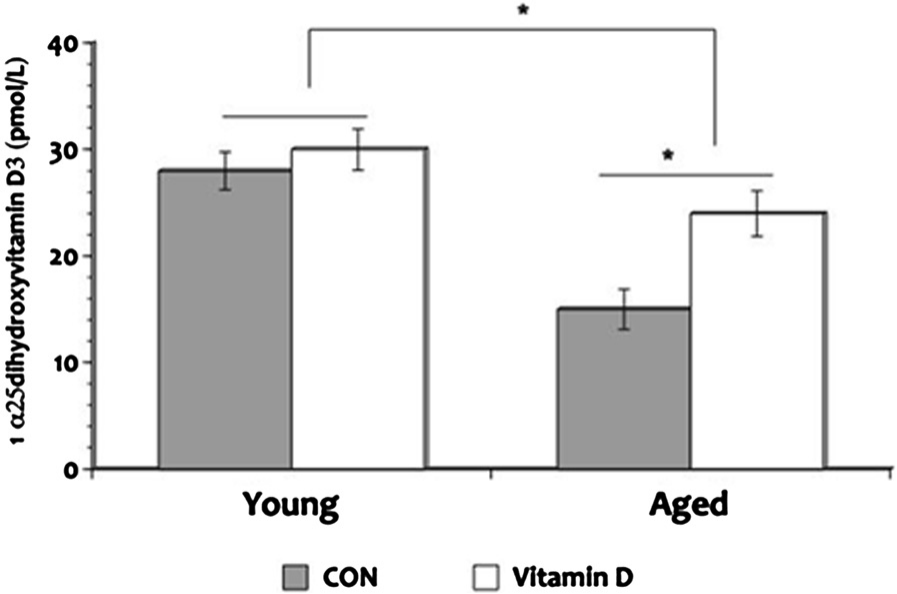
**Plasma levels of vitamin D.** Plasma circulating levels show significant deficiency in the aged rats when compared to the young animals in the active form of vitamin D. However, supplementation of vitamin D mitigated this deficiency. *P <0.05. Legend: CON, control.

To determine age-related vitamin D changes we measured circulating levels of its active form, vitamin D, 1, α25-dihydroxyvitamin D3, and found significantly lower levels in the aged rats when compared to the young animals (Figure 3); but the deficiency seen in the aged rats that received the supplementation was significantly less in comparison to the aged CON animals. Post hoc comparisons showed a significant 1, α25-dihydroxyvitamin D3 deficiency in the aged CON rats to less than 26% of the young CON group suggesting an age-related vitamin D deficiency. Further analysis revealed circulating levels of 1, α25-dihydroxyvitamin D3 is 32% in the aged CON rats compared to the aged animals that received the supplementation. No significant differences were evident in circulating levels of 1, α25-dihydroxyvitamin D3 in the young animal groups. These results suggest an age-related deficiency in vitamin D metabolism.

### Vitamin D mitigates age-related cognitive decline in the water maze task

**Figure 4:**
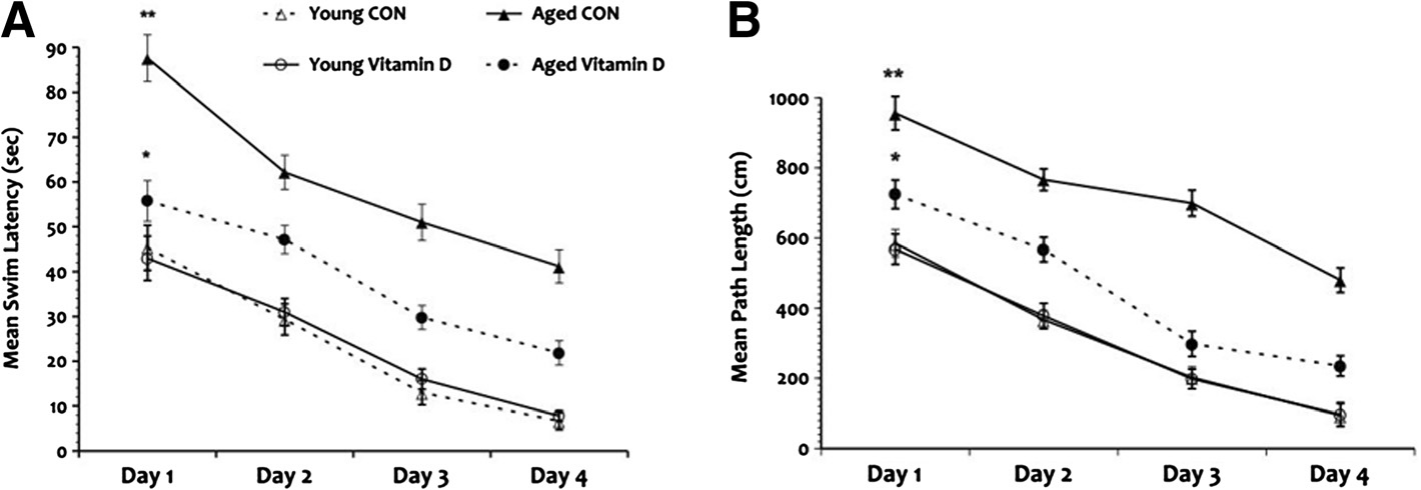
**Water maze task.** Performance in the water maze shows significant impairment in the aged rats in swim latency (**A**) in comparison to the young animals with the greatest deficit seen in the aged control group. Analysis of path taken to reach the goal (**B**) also show that the aged rats was significantly impaired compared to the young animal groups but the aged rats that received vitamin D supplementation performed slightly better. *P <0.05, **P <0.01. Legend: CON, control.

Rats were tested in the water maze after ensuring intact visual skills. A significant within-subjects effect was seen for swim latency across the test days given that all rats learned the task over the 4 testing days. An overall significant main effect of age was seen in mean swim latency (Figure 4A) and the aged CON group demonstrated longer mean swim latency overall compared to all groups. Meanwhile, a significant main effect of vitamin D was also seen in that the aged rats that received vitamin D demonstrated decrease mean swim latency compared to the aged CON group. Although the aged vitamin D group performed significantly better when compared to the aged CON rats, they still demonstrated impairment in comparison to the young animal groups. No significant difference was seen in mean swim latency between the young animal groups. Additionally, no interaction effect was seen between age and vitamin D in mean swim latency but a significant interaction effect was seen in swimming speed (*P* = 0.031) with longer times seen in the aged animal groups.

Measurement of path length also showed significant within-subjects effect (Figure 4B) where all rats showed a continuous decrease in the swimming distance covered to reach the platform across the test days. Overall significant main effects of age and vitamin D were seen where the aged CON animals frequently used the indirect path to reach the goal compared to all groups while the aged vitamin D group performed better but still showed impairment in comparison to the young animals. No significant difference was seen when the young animal groups were compared. A trend toward an interaction effect (*P* = 0.06) was seen between age and vitamin D in mean path length. Taken together, the swim latency and path length results suggest that the rats were able to determine their location in space, and adjust their behavior accordingly but this behavior just took longer to accomplish in the aged animals; however, giving vitamin D to the aged rats helped lessen this cognitive decline.

In the probe trial (long-term memory recall phase) time spent swimming in the quadrant of the pool where the goal/platform was previously located (correct quadrant) was divided by the time spent swimming in the other three quadrants of the pool (wrong quadrants). Aged CON rats showed significant impairment overall (Table 2) when compared to all groups. In contrast, aged rats that received vitamin D supplementation performed slightly better but still spent more time in the incorrect quadrant area and made more search errors when compared to the young animal groups. No significant differences were seen in the probe trial performance of the young rats. These results suggest that long-term memory recall is also affected by age and that vitamin D supplementation diminished this cognitive impairment.

**Table 2:** Behavioral performance in the Probe Trial

	**Errors (*n*)**	**Time spent in correct quadrant (%)**
Young CON	1.51 ± 0.27	62% ± 2.93
Young vitamin D	1.60 ± 0.32	65% ± 3.40
Aged CON	3.25 ± 0.21^a^	41% ± 3.17^a^
Aged vitamin D	2.49 ± 0.35^b^	52% ± 3.02^b^

Aged CON rats made more mistakes in the probe trial compared to all groups, while the aged rats that received vitamin D supplementation performed slightly better but still remained impaired when compared to the young rats.

^a^*P* <0.01.

^b^*P* <0.05.

### Vitamin D mitigates age-related cognitive decline in the spontaneous object recognition task

**Figure 5:**
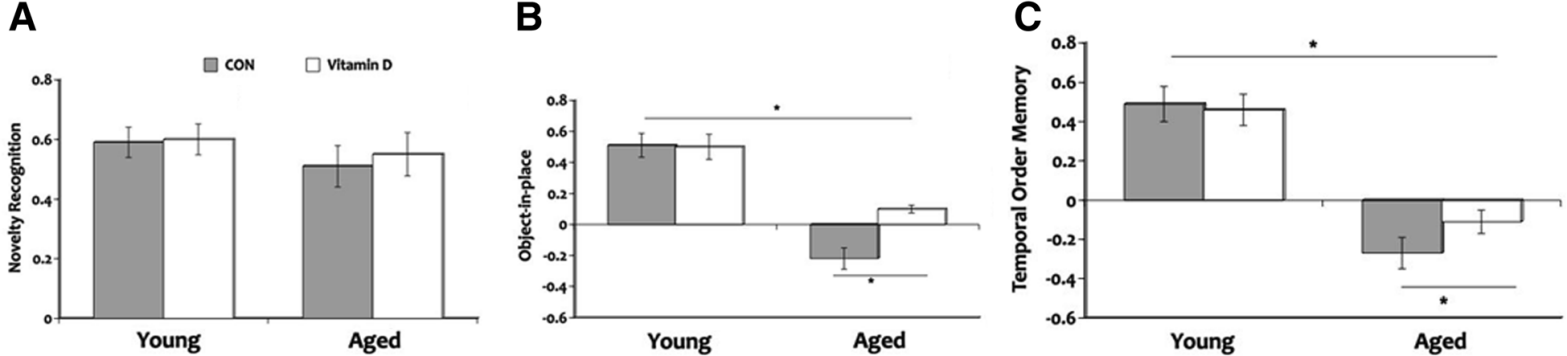
**Discrimination ratio in the spontaneous object recognition tasks.** Performances in the novelty object test (**A**) show no significant differences in all groups. However, aged rats showed significant impairment in discriminating between the objects presented in the object-in-place test (**B**) and temporal order memory test (**C**) compared to the young animals but vitamin D supplementation ameliorated this cognitive impairment. *P <0.05. Legend: CON, control.

Analysis of performance in the novelty recognition task showed no significant main effect of age and vitamin D in exploration times during the sample phase. Separate analysis of exploration times in the test phase also showed no significant group differences as seen in Table 3. Furthermore, discrimination ratio on the test phase showed no significant main effect of age nor vitamin D wherein all rats demonstrated the ability to discriminate between the familiar and novel objects even after a time delay between the sample and test phases (Figure 5A). As well, no significant interaction effect was seen between age and vitamin D in both total exploration times and discrimination ratio. These results suggest that hippocampal aging does not affect the animals’ ability to recognize simple novelty.

**Table 3:** Exploration time in the spontaneous object recognition tasks

	**Novel object preference**		**Object-in-place**	**Temporal order memory**
	**Sample phase**	**Test phase**	**Test phase**	**Test phase**
Young CON	49.7 ± 2.21	47.5 ± 3.01	31.1 ± 3.47	33.3 ± 2.51
Young vitamin D	48.2 ± 2.93	45.9 ± 2.99	34.4 ± 3.72	35.2 ± 1.88
Aged CON	47.8 ± 1.97	46.9 ± 3.15	23.7 ± 4.34^a^	20.1 ± 2.43^a^
Aged vitamin D	49.3 ± 2.10	47.5 ± 3.07	29.9 ± 4.40	11.9 ± 2.16^b^

Mean exploration time expressed in seconds(s) during the sample and test phases in the novelty recognition test, and during the test phases in the object-in-place and temporal order memory tests. No significant differences were seen in exploration time during the novelty recognition and object-in-place tests. Meanwhile, aged CON rats showed significantly decrease exploration time in the object-in-place and temporal order memory tests after a time delay.

^a^*P* <0.05 (significantly different from the young animal groups). **p <0.01 (significantly different overall).

^b^*P* <0.01 (significantly different overall).

When performance was evaluated in the object-in-place task, our results showed that exploration time in aged animals was not significantly different from the young rats after a 30 min delay (Table 3). However, significant main effects of age and vitamin D were seen in discrimination ratio where it is lower in aged rats compared to the young animals (Figure 5B) but aged rats that received vitamin D supplementation showed slightly better performance when compared to the aged CON rats. Comparison of the young animals showed no significant difference in discrimination ratio in the object-in-place task. These results suggest that aged animals lacked the ability to differentiate between the non-rearranged and rearranged objects but vitamin D was able to mitigate this impairment.

Analysis of recognition in the temporal order memory test after a 90-min delay revealed a significant main effects of age and vitamin D in exploration time as seen in Table 3. Post hoc testing confirmed that exploration time of the aged control animals was significantly worse when compared to all groups. But aged rats given vitamin D supplements showed improvement in exploration time when compared to the aged CON animals even though their performance is still impaired in comparison to the young rats. No significant difference was seen in exploration time between the young animal groups. Analysis of discrimination ratio showed that aged rats were less able to discriminate between the objects presented earlier and the one presented recently when compared to the young animal groups (Figure 5C). Although aged rats that received vitamin D supplements failed to significantly discriminate between the objects in the temporal order memory task when compared to the young rats, their performance is not as worse as the aged CON animals. These results suggest the possibility that vitamin D may be able to ameliorate age-related cognitive decline in distinguishing complex stimuli.

### Vitamin D modulates the age-related changes in inflammatory state

**Figure 6:**
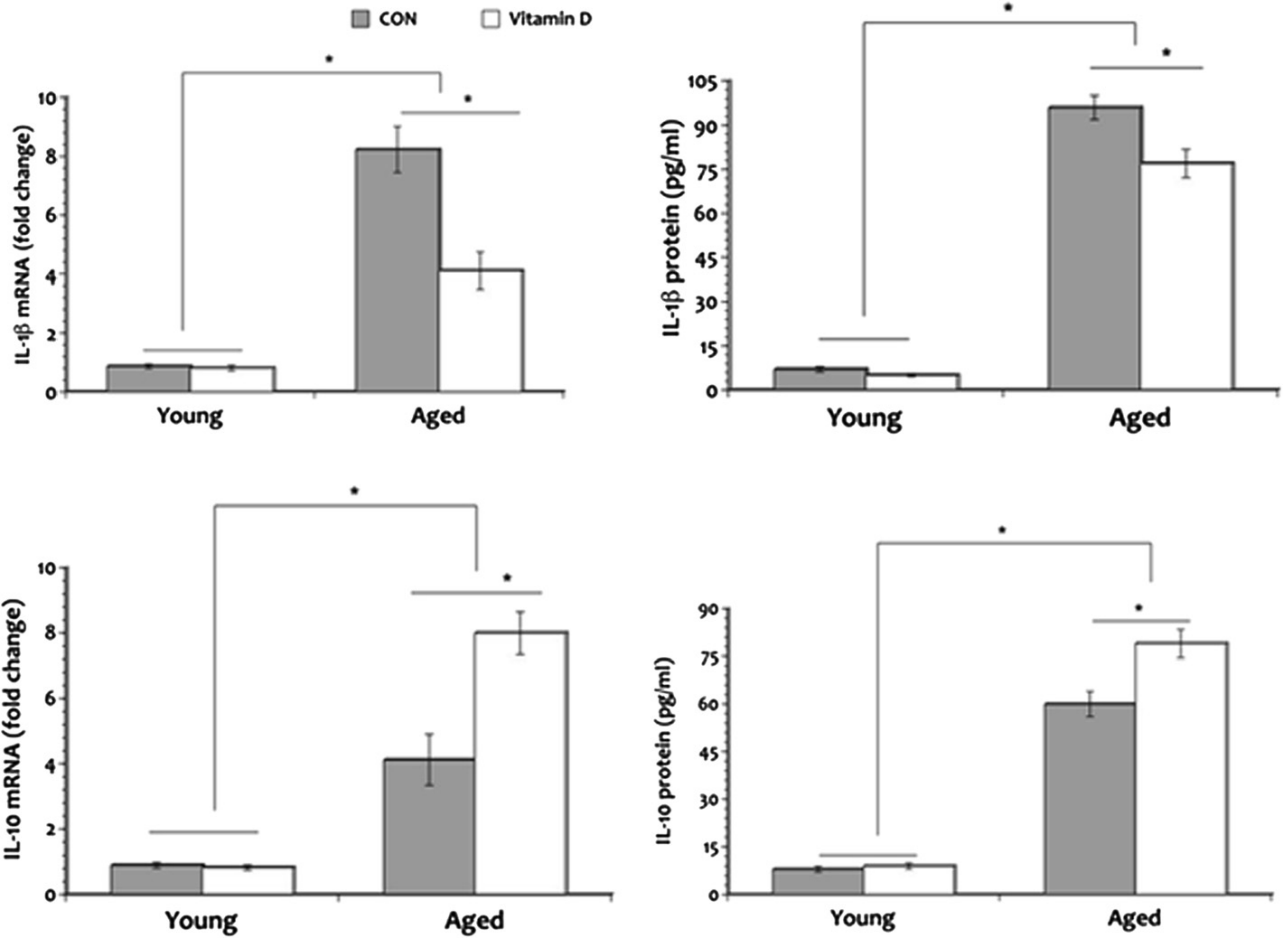
**IL-1β and IL-10 expression.***Upper panel*: IL-1β expression. *Lower panel*: IL-10 expression. A significant elevation in IL-1β mRNA and protein expression was seen in the aged rats in comparison to the young animals but vitamin D supplementation mitigated the increase seen in this pro-inflammatory cytokine in the hippocampus. Levels of IL-10 mRNA and protein also increased in aging as compared to the young rats but vitamin D supplementation significantly augmented the age-related increase in this anti-inflammatory cytokine. *P <0.05. Legend: CON, control.

To determine whether vitamin D can improve the balance between pro- and anti-inflammatory states that may be disrupted in aging, we examined mRNA and protein levels of IL-1β and IL-10 in the hippocampus. Results reveal that both aged groups have significantly increased levels of the pro-inflammatory cytokine IL-1β mRNA when compared to the young animals (Figure 6A) with aged CON rats demonstrating an overall higher levels. Levels of IL-10 mRNA is also significantly elevated in both aged groups compared to the young rats but IL-10 mRNA levels in aged rats that received vitamin D supplementation was greater in comparison to the aged CON group. Similar pattern of expression is seen in IL-1β and IL-10 protein expression. Furthermore, analyses of IL-1β and IL-10 protein levels (Figure 6B) show similar pattern of expression as those seen in mRNA. Both young animal groups show minimal expression of IL-1β and IL-10 mRNA as well as protein in the hippocampus and no vitamin D effect is seen. These results suggest that vitamin D may be able to restore the disrupted equilibrium between the pro- and anti-inflammatory states seen in aging.

### Vitamin D facilitates Aβ clearance and decrease amyloid burden

**Figure 7:**
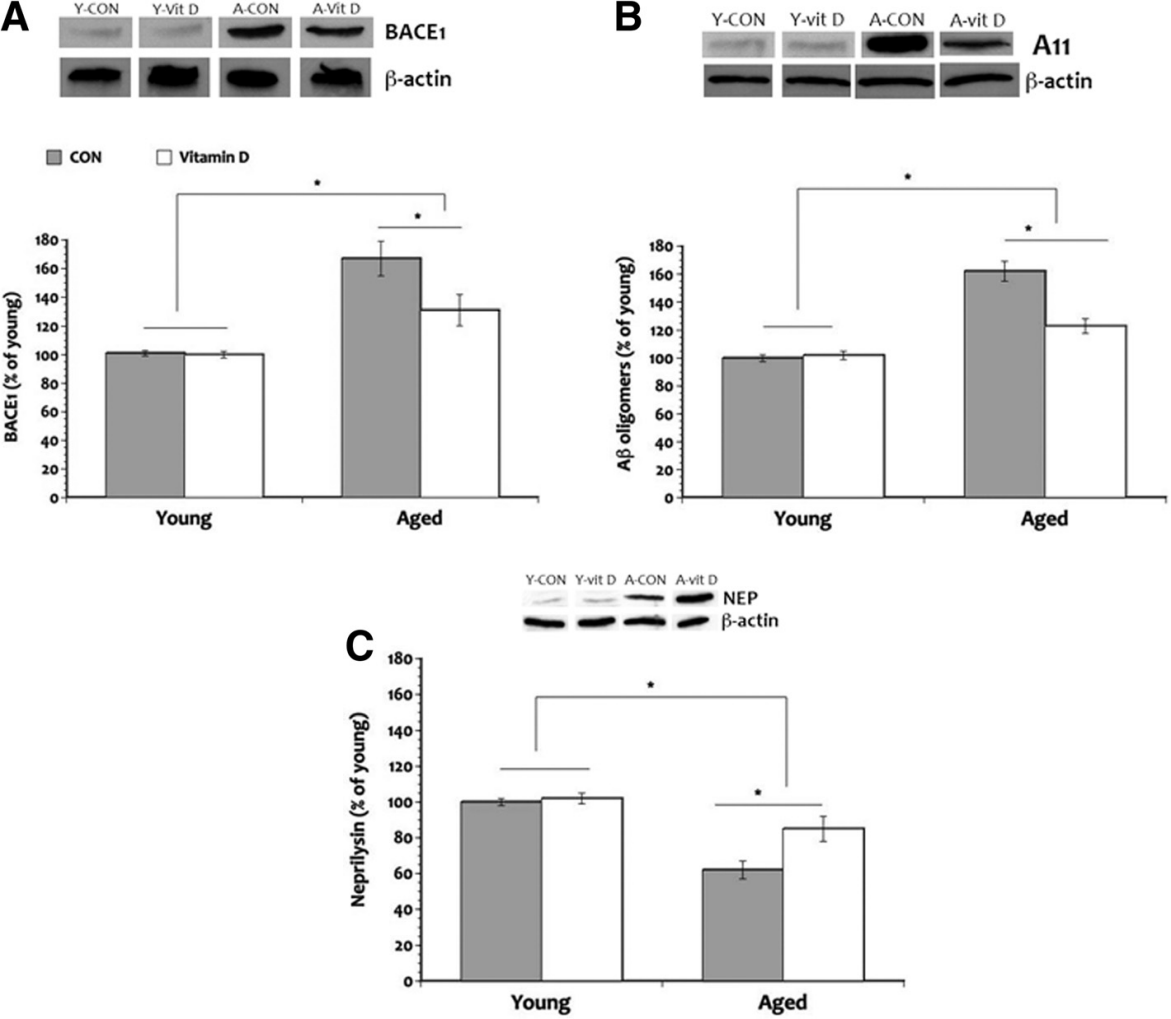
**Beta amyloid clearance.** Significant elevation of β-secretase amyloid cleaving enzyme (BACE1) level (**A**) led to increased production of Aβ oligomers (**B**) in the aged rats when compared to the young animals. Moreover, the significantly reduced levels of neprilysin (NEP), classical amyloid-degrading enzyme (**C**), in the aged control rats when compared to the aged vitamin D group possibly decreased amyloid clearance. Nonetheless, the development of this age-related neuropathology was abated by vitamin D supplementation. *P <0.05. Legend: A, aged; CON, control; vit, vitamin; Y, young.

We examined whether age-related neuroinflammation is associated with increased formation of Aβ oligomers and if this amyloid oligomerization results from either: (1) increased alternative processing of the amyloid precursor protein through the amyloidogenic pathway; or (2) decreased clearance of beta amyloid. Increased alternative processing of the amyloid precursor protein through the amyloidogenic pathway is initiated by the β-secretase amyloid cleaving enzyme (BACE) and can lead to the accumulation of Aβ oligomers. We observed significant increase in the formation of amyloid oligomers in the hippocampus of aged rats (35% and 18% greater in the aged CON and aged vitamin D groups, respectively) compared to the young animals (Figure 7B). However, giving vitamin D supplementation significantly reduced Aβ oligomerization by 22% when compared to the aged CON. No significant difference in the formation of Aβ oligomers was seen in the young animals. These results suggest that vitamin D was able to lessen the formation of the toxic form of the Aβ peptide commonly associated with aging.

Additional analysis revealed that the pattern of BACE1 expression seen in the aged rats is similar to that of Aβ oligomers (Figure 7A) further confirming that aging may be related to increased processing of the full-length amyloid precursor protein by the amyloidogenic or alternative pathway facilitated by the β-secretase complex, thus generating more amyloid peptides. Vitamin D supplementation on the other hand, was able to modulate BACE1 expression in the aged rats. BACE1 expression in the aged vitamin D animals was approximately 24% lower than the aged CON animals. But our data did not reveal any discernable impact on BACE1 expression in the young rats.

To assess the possibility that the reduction in the formation of Aβ oligomers seen in the aged rats that received vitamin D may also be a result of increased degradation or clearance of beta amyloid, we examined levels of the classical Aβ-degrading enzyme neprilysin (NEP) in the hippocampus. Our results revealed a significant main effect of age in that aged rats show decreased NEP levels (28% and 17% less in the aged CON and aged vitamin D, respectively) compared to the young animals (Figure 7C). A significant main effect of vitamin D is also seen in older rats that received the supplement where NEP levels significantly increased (15% more) compared to the aged CON animals. No significant difference was seen in levels of NEP in the young animal groups. These results suggest that the activity of the Aβ degradation pathway mediated by NEP decrease with age and that providing vitamin D supplementation may be helpful in improving this age-related change in amyloid clearance.

## Discussion

In the present study we demonstrate that age-related vitamin D deficiency is associated with enhanced pro-inflammatory state, increased formation of Aβ oligomers, and reduced amyloid clearance in the hippocampus and that these neuropathological processes are accompanied by cognitive decline. To our knowledge this is the first study to link the neuro-immune effects of vitamin D in the normal aging brain. Vitamin D is an important hormone with well-characterized effects in the whole body system in early life as well as in later life. In the past decades, our knowledge about vitamin D and its biological activity has significantly improved. Reports on vitamin D deficiency in the elderly [1,12,13] is extended in the findings of the present study where we show that aged animals are deficient in the active form of vitamin D, 1, α25-dihydroxyvitamin D3, when compared to the young animals. The age-related deficiency in vitamin D demonstrated here may be due to decrease production and/or increase metabolic clearance rate because of the high levels of 24-hydroxylase, the enzyme that metabolize vitamin D, seen in the elderly (reviewed in [30]). The lack of significant difference in circulating levels of 1, α25-dihydroxyvitamin D3 in the young animals is hard to explain but it may be that the amount of supplement used in the present study is not high enough to cause an increase in plasma levels in conditions where no deficiency exist.

Biological effects of vitamin D supplementation in aging are illustrated in our findings. Here we demonstrate an age-related disruption in the homeostatic balance between the common pro- and anti-inflammatory cytokines, which parallel findings from previous reports [22,24,31,32]. We also show that supplementation with the active form of vitamin D modulated the age-related changes in pro-inflammatory state where the anti-inflammatory cytokine IL-10 increased while the pro-inflammatory cytokine IL-1β decreased. Because vitamin D plays a regulatory role in inflammation (reviewed in [30]), its deficiency can promote a chronic pro-inflammatory state that can have deleterious effects in the brain. For example, the pro-inflammatory cytokine IL-1β plays a role in amyloid precursor protein processing and promotes Aβ production [33]. Increase Aβ production then results in secretion of more pro-inflammatory cytokines, thus augmenting amyloid toxicity [34]. Indeed, our study show that the upregulation in IL-1β seen in the aged CON rats is accompanied by increase production of the ‘toxic’ Aβ oligomers through the processing of the amyloid precursor protein via the amyloidogenic pathway facilitated by BACE; and this is confirmed by our data that BACE1 levels is elevated in the hippocampus of the aged CON rats.

The immune system’s innate response to curb chronic inflammation is possibly mediated by vitamin D by regulating the release of anti-inflammatory cytokines such as IL-10. The role of IL-10 in controlling and terminating CNS inflammation has been widely reported [35] as well as its ability to inhibit Aβ effects [36]. Data from the present study extend previously reported findings [35,36] where we show that significantly increased IL-10 expression in the aged rats that received vitamin D is associated with a significant decreased in IL-1β suggesting possible restoration in the homeostatic balance of inflammatory mediators that may be disturbed in aging. An unexpected finding is the significant increase in IL-10 levels in the aged CON rats compared to the young animal groups and a possible explanation for this is that it is a compensatory mechanism for the increased pro-inflammatory state associated with aging to counterbalance the increase in IL-1β. The vitamin D-mediated upregulation of IL-10 in the aged rat brain is particularly important because it possibly mediated the reduction in risk for developing neuropathology by decreasing BACE1 levels and consequent formation of Aβ oligomers.

The mechanism whereby vitamin D regulates Aβ burden is most likely related to its ability to regulate amyloid clearance. Balance between the synthesis of Aβ from the amyloid precursor protein and the removal of this peptide by amyloid-degrading enzymes is an important factor in determining amyloid clearance preventing its accumulation thereby decreasing Aβ burden. It has been suggested that accumulation of Aβ may be explained by a reduction in the catabolic activity of Aβ-degrading enzymes such as NEP. Here we show that NEP level is significantly decreased in the aged rats compared to the young rats but this reduction in amyloid degrading enzyme is mitigated by vitamin D supplementation. The decreased catabolic activity of amyloid-degrading enzymes seen in aging may be due to the system being overwhelmed by the increased production of Aβ so that NEP levels were depleted faster compared to the young rats.

Taken together, our data show that the modulation of the pro-inflammatory state after vitamin D supplementation in the aged animals is associated with minimizing the pathological consequences of neuroinflammation evidenced by the reduction of BACE1 level and the consequent reduction in Aβ oligomer formation. Aβ is a regulatory peptide produced during normal brain metabolism and its steady-state concentration is tightly controlled by amyloid-degrading proteolytic enzymes [37]. The combination of increased Aβ formation and decreased amyloid-degrading enzyme seen in aging can lead to an elevated amyloid burden. Since our data show that vitamin D supplementation in aging can both minimize the formation of Aβ oligomers and increase the activity of the amyloid-degrading enzyme NEP, it stands to reason that this hormone can be neuroprotective by reducing amyloid burden and facilitating amyloid clearance.

Vitamin D is also implicated in regulating behavioral functions. In the present study we examined vitamin D effects of a broad range of memory processes. In this study we show an attenuation of cognitive deficits in the aged rats given vitamin D supplements compared to the aged CON group when tested in the water maze. The two key findings are: (1) decreased mean swim latency and path taken to reach the goal in the aged vitamin D group when compared to the aged CON group; and (2) increased memory recall during the probe trial in the aged rats given vitamin D in comparison to the aged CON animals. Although all rat groups seemed to exhibit the ability to learn the task of reaching the goal, the aged CON rats persistently show increased time and longer path taken to reach the goal, as well as decreased ability to remember the previous location of the goal; however, age-related impairment in the water maze performance is diminished by vitamin D supplementation suggesting that this neurohormone may be able to attenuate aged-related memory decline. Of the behavioral measures used in the water maze, it is possible that mean swim latency may be the least sensitive. That is, the increased swim latency demonstrated by the aged vitamin D group may be more related to age-related decrease in motoric activity and not an indication of cognitive decline. This line of reasoning is supported by our data that the aged vitamin D group took a more efficient path to reach the goal when compared to the aged CON animals and that we did see a significant difference in swimming speed.

Spontaneous object recognition memory is a more complex test that uses information such as relative familiarity of an object or location or when or where an object is previously encountered to make decisions. Spontaneous object recognition tasks involve not only hippocampal functioning but also the medial prefrontal cortex as well as the perirhinal cortex. In the present study we demonstrate that aging had no influence on an animal’s ability to recognize object novelty. But the rat’s recency memory and ability to recognize the rearrangement of objects previously encountered are impaired evidence by significantly decrease discrimination ratio in the object-in-place and temporal order memory tests. Nevertheless, vitamin D supplementation ameliorated the age-related impairment in object-in-place and temporal order memory tasks. The impairments in the object-in-place and temporal order memory tasks are most likely due to functional alterations in the medial prefrontal and perirhinal cortices, and the hippocampus, which probably occur during aging leading to increased vulnerability to the interfering effects of stimuli encountered during a delay period. The increased vulnerability to interference may be due to the inability to distinguish between complex stimuli that share common features. Since vitamin D receptors are widely distributed in the cortex and hippocampus [3-5], it is possible that the supplementation allowed more circulating hormone to exert its biological function thereby minimizing cognitive decline.

## Conclusion

In sum, the results presented in this study demonstrate that vitamin D deficiency is common in aging and that low levels of vitamin D seen in the aged rats are associated with increased odds of cognitive impairment. Supplementation of vitamin D was able to improve age-related cognitive decline and the possible mechanism whereby this neurohormone exert its beneficial function is by modulation of immune function by restoring the balance between pro- and anti-inflammatory cytokine secretions, which may be lost in aging. By controlling pro-inflammatory cytokine secretion, vitamin D minimizes the risk for neuropathological consequences such as decreasing Aβ burden and increasing amyloid clearance. The amount of vitamin D supplement used in the study did not completely reverse age-related changes to the point that both behavioral and biochemical measures return to levels comparable to the young animal groups but our results demonstrate that it is more than adequate to minimize functional deficits and amplification of the vulnerability in developing neuropathology associated with aging. Although this study does not prove a causal relationship between vitamin D and neurological function, the data presented suggest an immense potential for this neurohormone to modify age-related impairment in cognitive and biological functioning. Given that neurodegeneration and cognitive impairment are likely to play a central role in the healthcare system and society in the near future, correction of vitamin D deficiency is an easy, inexpensive, and safe way to modify the risk factors associated with aging.

## Competing interests

The authors declare that they have no competing interests.

## Authors’ contributions

HD conceived of the study and participated in its design. TLB participated in the study design and performed the experiments, statistical analyses, as well as drafted the manuscript. All authors read and approved the final manuscript.
